# Supplementary material for: Saccharomyces boulardii (CNCM I-745) improves intestinal damage in sepsis by remodeling intestinal flora structure
Source: Front Cell Infect Microbiol. 2025 Jul 31;15:1602792. doi: 10.3389/fcimb.2025.1602792 (PMC12350329; doi:10.3389/fcimb.2025.1602792)
Supplement: Supplementary file 1 [file DataSheet1.zip › summary/summary/6_Taxonomy_community/1_Taxonomy_profiling/5_Family/Venn/venn_diagram_Group.DW0_vs_CW0_vs_BW0_vs_AW0.pdf]

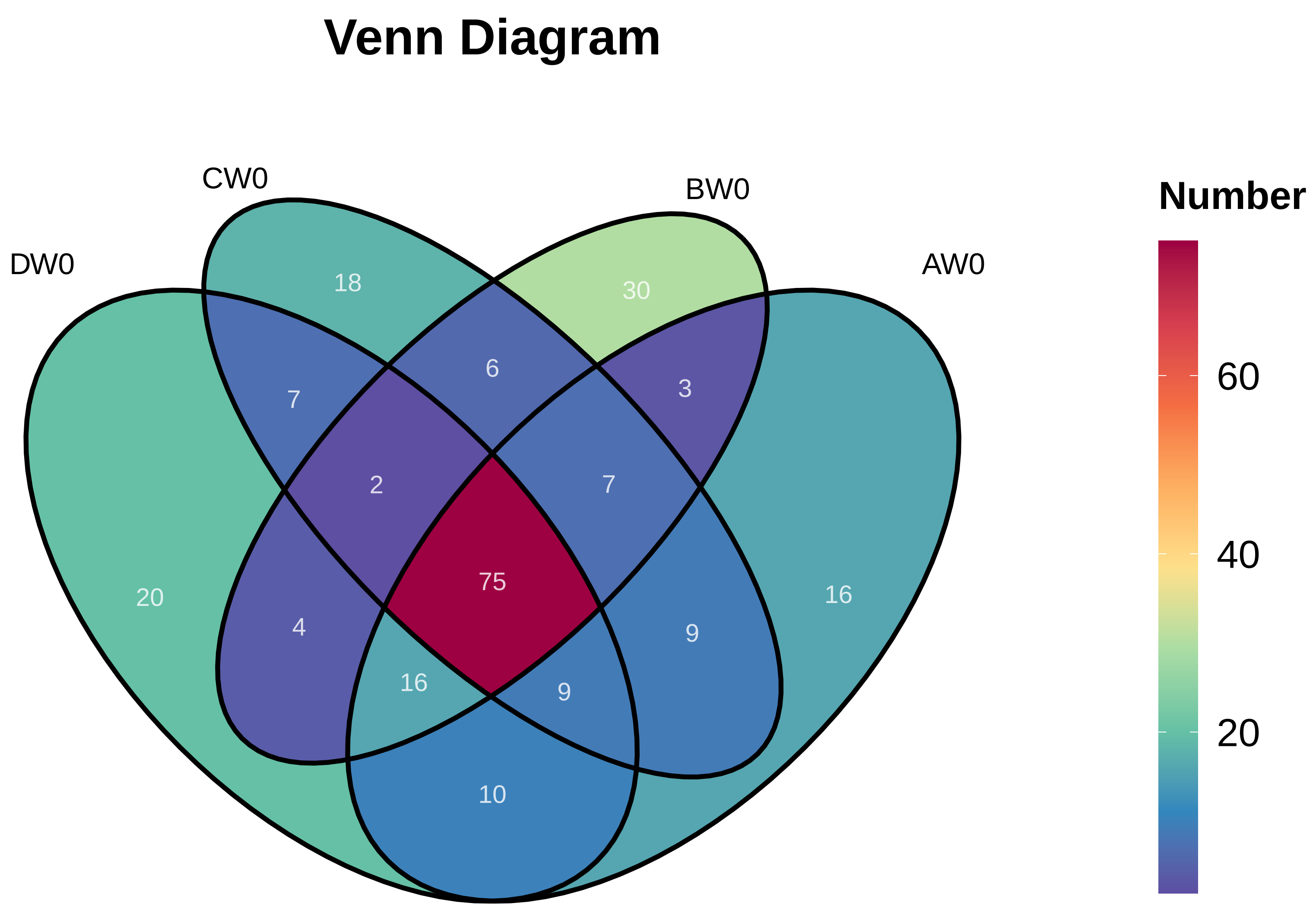
Venn Diagram
DW0
CW0
BW0
AW0
18
30
7
6
3
2
7
20
75
16
4
9
16
9
10
Number
60
40
20
